# miR-224-5p Contained in Urinary Extracellular Vesicles Regulates PD-L1 Expression by Inhibiting Cyclin D1 in Renal Cell Carcinoma Cells

**DOI:** 10.3390/cancers13040618

**Published:** 2021-02-04

**Authors:** Zhiyuan Qin, Haihong Hu, Wen Sun, Lu Chen, Shengnan Jin, Qingwen Xu, Yuxi Liu, Lushan Yu, Su Zeng

**Affiliations:** 1 Institute of Drug Metabolism and Pharmaceutical Analysis, College of Pharmaceutical Sciences, Zhejiang University, Hangzhou 310058, China; qinzy20@zju.edu.cn (Z.Q.); huhaihong@zju.edu.cn (H.H.); sunw127@zju.edu.cn (W.S.); 11419030@zju.edu.cn (L.C.); snjin@zju.edu.cn (S.J.); 11819035@zju.edu.cn (Q.X.); liuyuxi@zju.edu.cn (Y.L.); 2 Zhejiang Province Key Laboratory of Anti-Cancer Drug Research, Cancer Center of Zhejiang University, Hangzhou 310058, China

**Keywords:** extracellular vesicles, renal cell carcinoma, miR-224-5p, cyclin D1, PD-L1

## Abstract

**Simple Summary:**

The detailed effects of abundant microRNAs contained in extracellular vesicles (EVs) on renal cell carcinoma (RCC) progression are still unclear. This study identified the overexpression of miR-224-5p in urinary EVs of RCC patients. miR-224-5p suppressed RCC cell proliferation and induced cell cycle arrest through inhibiting cyclin D1 expression. PD-L1 protein abundance was increased by miR-224-5p, and this regulation could be transmitted via EVs intercellularly. These findings may shed light on biomarker discovery for RCC immunotherapy.

**Abstract:**

The abundant miRNAs in urinary extracellular vesicles (EVs) represent ideal reservoirs for biomarker discovery, especially in renal cell carcinoma (RCC). However, the content and biological functions of microRNAs contained in urinary EVs in RCC remain ambiguous. In this study, urinary EVs were isolated and characterized from RCC patients and healthy volunteers. Differentially expressed microRNAs in urinary EVs were screened by small RNA sequencing. The target gene and biological functions of selected microRNAs were investigated through multifaceted methods. Results indicated that miR-224-5p was significantly upregulated in urinary EVs of RCC patients compared to healthy volunteers. The overexpression of miR-224-5p inhibited RCC cell proliferation and induced cell cycle arrest. The gene *CCND1* encoding cyclin D1 was identified as a direct target of miR-224-5p via prediction and validation. Moreover, the invasive and metastatic abilities of RCC cells were enhanced by miR-224-5p. Interestingly, miR-224-5p also increased the stability of PD-L1 protein by inhibiting *CCND1*. This effect could be transmitted via EVs and further promoted the resistance of RCC cells to T cell-dependent toxicity. In summary, urinary EVs containing miR-224-5p were identified as a potential biomarker in RCC. Regulation of PD-L1 protein expression by miR-224-5p through suppressing *CCND1* elucidates new roles of miR-224-5p in RCC progression.

## 1. Introduction

Renal cell carcinoma (RCC) is among the most common urological cancers, accounting for approximately 90% of all kidney cancer cases [1]. Owing to advances in early diagnosis techniques and targeted therapies, the five-year survival rate of RCC patients has improved in recent years. However, it is estimated that approximately one-third of patients with RCC are diagnosed with metastases, which typically require systemic treatments targeting tumor signaling pathways or the immune system [2]. One of the immunotherapies that target immune checkpoints, represented by blockade of programmed cell death protein 1 (PD-1) and its ligand programmed death ligand 1 (PD-L1), has been approved for clinical treatment of RCC patients [3]. Nevertheless, the response rate of PD1/PD-L1 inhibitors in RCC is relatively low [4]. Therefore, it is necessary to identify relevant PD-1/PD-L1 biomarkers to predict treatment responses.

A typical biomarker for PD-1/PD-L1 blockade strategies is the expression level of PD-L1 in tumors [5]. However, association between PD-L1 abundance and responses to immune checkpoint inhibitors in RCC remains unclear. Furthermore, the underlying mechanism of PD-L1 expression in RCC also need to be elucidated. Hence, it is necessary to clarify the mechanistic pathways that regulate PD-L1 expression, which may provide a molecular basis to improve the clinical response rate of PD-1/PD-L1 blockade across cancer types. Previous studies have shown that PD-L1 expression can be regulated at both transcriptional and post-transcriptional levels [6]. Recently, Zhang et al. reported that cyclin D and its catalytic partner of cyclin-dependent kinases (CDK) 4, as well as the cullin 3-speckle type POZ protein (SPOP)-E3 ligase are involved in the regulation of PD-L1 expression via proteasome-mediated degradation in various human cancer cell lines. This mechanism results in fluctuation of PD-L1 protein abundance during cell cycle progression [7]. However, there is still no research focusing on the effects of cell cycle progression on PD-L1 expression in RCC cell lines. Hence, it is valuable to investigate whether this mechanism may be effective in RCC cells.

Extracellular vesicles (EVs) are membrane-enclosed particles released from almost all kinds of cells, and which mediate intercellular communication via delivering bioactive molecules including proteins, lipids and nucleic acids. Due to their nanoscale dimensions and a bilayer lipid membrane that appropriately protect cargoes from degradation, EVs can stably exist in blood, urine, saliva and other types of bodily fluids. The multifaceted functions of EVs in RCC progression and potential applications in RCC treatment were recapitulated in our previous review [8]. microRNAs (miRNAs) are a class of small non-coding RNAs with 19–25 nucleotides in length. As one of the most abundant components of EVs, miRNAs can be loaded into EVs within donor cells and delivered to recipient cells, resulting in the induction of phenotypic effects [9]. Previous studies have summarized the roles of miRNAs in regulating PD-L1 expression [10,11]. However, to the best of our knowledge, whether regulation of PD-L1 expression by miRNAs can be transmitted by EVs remains unknown. In the present study, we speculate that miRNAs in urinary EVs may be involved in the regulation of PD-L1 in RCC cells. Multifaceted methods, including RNA sequencing, bioinformatics tools, and in vitro functional experiments, were utilized to identify the bioactive molecules. Hence, findings from our study will shed light on mechanisms involved in PD-L1 expression and offer a molecular basis for biomarker discovery for immunotherapy in RCC.

## 2. Results

### 2.1. Isolation and Characterization of EVs from Human Urine

**Figure 1:**
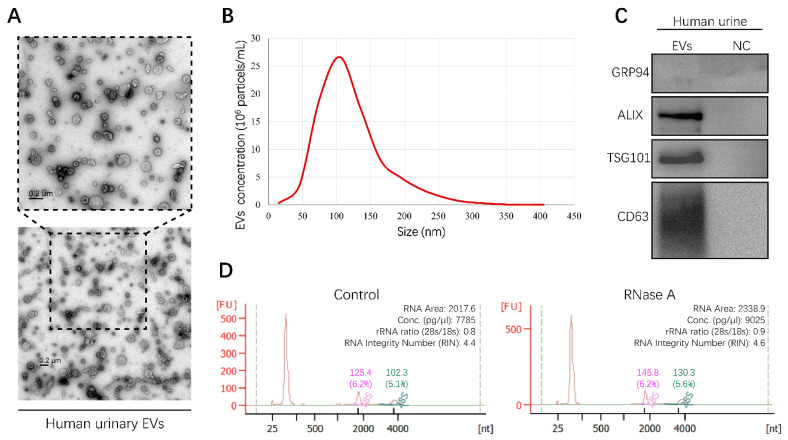
Characterization of human urinary extracellular vesicles (EVs). All experiments were performed as three independent experiments, and representative results were selected. (**A**) Transmission electron microscopy (TEM) images of EVs isolated from human urine sample (Scale bar, 200 nm). (**B**) The size and concentration of EVs were determined by nanoparticle tracking analysis (NTA). (**C**) Specific protein marker identification of EVs was conducted by Western blot analysis. Supernatants of last ultracentrifugation of each EVs sample were used as negative control (NC). (**D**) RNA landscapes of human urinary EVs with or without RNase A treatment were profiled by an Agilent 2100 Bioanalyzer. FU, fluorescence intensity. nt, RNA length in nucleotides. (Original Western blots see Figure S7).

In order to obtain urinary EVs containing miRNAs, EVs derived from human urine samples were isolated by differential ultracentrifugation (UC) in this study. To characterize the shape and size distribution of EVs, EVs were examined by transmission electron microscopy (TEM) and nanoparticle tracking analysis (NTA), respectively. The typical cup-shaped morphology of EVs ranging from 30 to 150 nm in diameter was observed by TEM (Figure 1A). The results of NTA displayed a size distribution consistent with TEM observations (Figure 1B). Furthermore, positive EV protein markers, including ALIX, TSG101, and CD63, were identified in urinary EVs through Western blot analysis, while a negative protein marker, GRP94, was detected faintly (Figure 1C). These results provided direct evidence that human urinary EVs could be classified as exosomes [12]. In addition, characterization of EVs derived from the culture medium of RCC cell lines was described in our previous study [13]. To investigate the integrity of EVs and exclude contamination of cell-free RNA, human urinary EVs were incubated with or without RNase A for 1 h at 37 °C. RNA was then extracted and assessed using the Agilent 2100 Bioanalyzer system. The results showed that RNase A treatment had no effect on the amount and fragment size distribution of small RNAs packaged in EVs (Figure 1D). Altogether, the method established in this study could be used to isolate integral EVs from the human urine samples.

### 2.2. miRNA Expression in Human Urinary EVs

**Figure 2:**
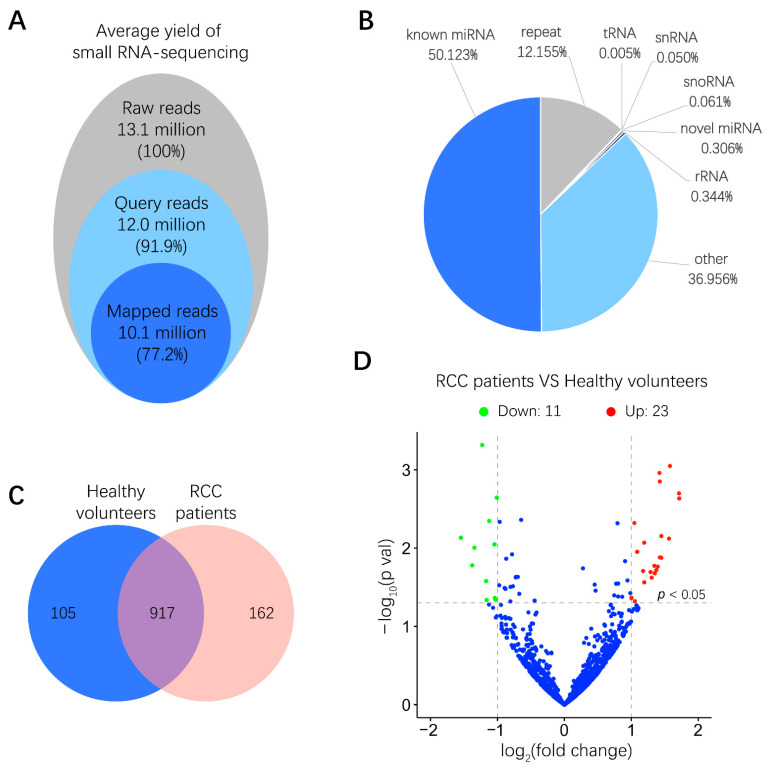
Small RNA sequencing results of miRNA expression levels in human urinary EVs. (**A**) Overview of small RNA-sequencing results including the number and percentage of total reads, query reads and mapped reads. (**B**) Distributions of different non-coding small RNA types in mapped reads. (**C**) Venn diagram of identified common and unique miRNAs in RCC patients and healthy volunteers. (**D**) Volcano plot of differences between miRNAs in urinary EVs were classified according to the fold changes (log_2_ (fold change)) between RCC patients (n = 6) and healthy volunteers (n = 6). Green, red and blue dots mean that miRNAs expression have significant downregulation, upregulation and no significant difference in RCC patients compared with healthy volunteers, respectively (|log_2_ (fold change)| > 1, *p* < 0.05).

To examine the miRNA expression pattern in urinary EVs from six RCC patients and six healthy volunteers, total RNA derived from urinary EVs was isolated, and the quality of RNA samples was assessed by a microfluidics-based electrophoresis analysis. A small RNA sequencing platform was utilized to profile miRNAs exhibiting differential expression between RCC patients and healthy volunteers. After low-quality reads and adapter contamination were eliminated, around 10 million mapped reads were acquired for each sample on average (Figure 2A). Results indicated that more than 50% reads were known miRNAs that could be aligned to the miRBase database (http://www.mirbase.org/) (Figure 2B). As shown in Figure 2C, totally 917 miRNAs were identified in both RCC patients and healthy volunteers. miRNAs expressing a more than 2-fold change, *p* < 0.05, between RCC patients and healthy volunteers were further screened out, among which the abundance of 11 and 23 miRNAs was significantly lower and higher in RCC patients than in healthy volunteers, respectively (Figure 2D and Figure S1). These miRNAs with differential abundance in urinary EVs from RCC patients may provide valuable information for biomarker discovery.

### 2.3. Overexpression of miR-224-5p in RCC

**Figure 3:**
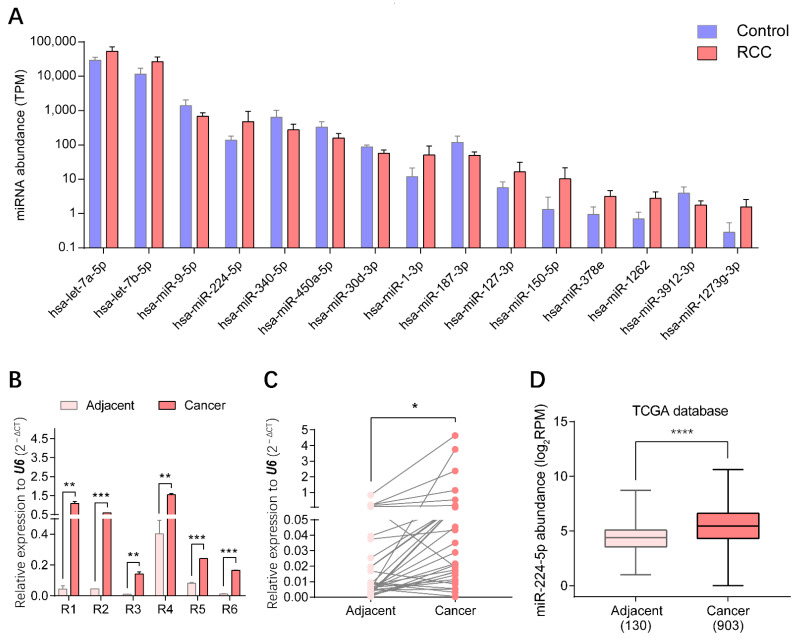
miR-224-5p was overexpressed in both urinary EVs and cancer tissues of RCC patients. (**A**) Top 15 significantly expressed miRNAs in urinary EVs from RCC patients and healthy volunteers (*p* < 0.01). (**B**) miR-224-5p levels in cancer and adjacent tissues of RCC samples used in small RNA-sequencing were determined by RT-qPCR. Data are mean ± s.e.m. (**C**) miR-224-5p expression levels in paired-tissues of RCC patients were determined by RT-qPCR (n = 35). (**D**) miR-224-5p expression levels in TCGA database (n = 130 for adjacent group; n = 903 for cancer group). * *p* < 0.05; ** *p* < 0.01; *** *p* < 0.001; **** *p* < 0.0001.

Through small RNA sequencing of urinary EV samples, the top 15 miRNAs presenting statistically significant differential expression in RCC patients compared with healthy volunteers (*p* < 0.01) were identified, as shown in Figure 3A. Three upregulated miRNAs, miR-1-3p, miR-150-5p and miR-224-5p, were screened to further validate the expression patterns in RCC. The levels of these candidate markers in cancer and adjacent tissues of RCC patients were determined by reverse transcription-quantitative PCR (RT-qPCR), correspondingly. Results indicated that levels of miR-224-5p were significantly upregulated in cancer tissues compared to paired adjacent tissues of six RCC patients whose urinary samples were used for EV isolation, which was consistent with the results of urinary EVs profiled by small RNA sequencing (Figure 3B). Furthermore, there was a similar trend for another 35 paired tissue samples of RCC patients (Figure 3C). Data mining results from The Cancer Genome Atlas (TCGA) database also revealed that miR-224-5p levels were markedly higher in cancer tissues than that in adjacent tissues of RCC patients (Figure 3D). However, expression levels of the other two miRNA candidates (miR-1-3p and miR-150-5p) in tissues were inconsistent with RNA sequencing results (Figure S2). Altogether, the overexpression of miR-224-5p in RCC tissues and urinary EVs will provide convincing clues for its potential as a biomarker for RCC.

### 2.4. miR-224-5p Induced Cell Cycle Arrest in RCC Cells

**Figure 4:**
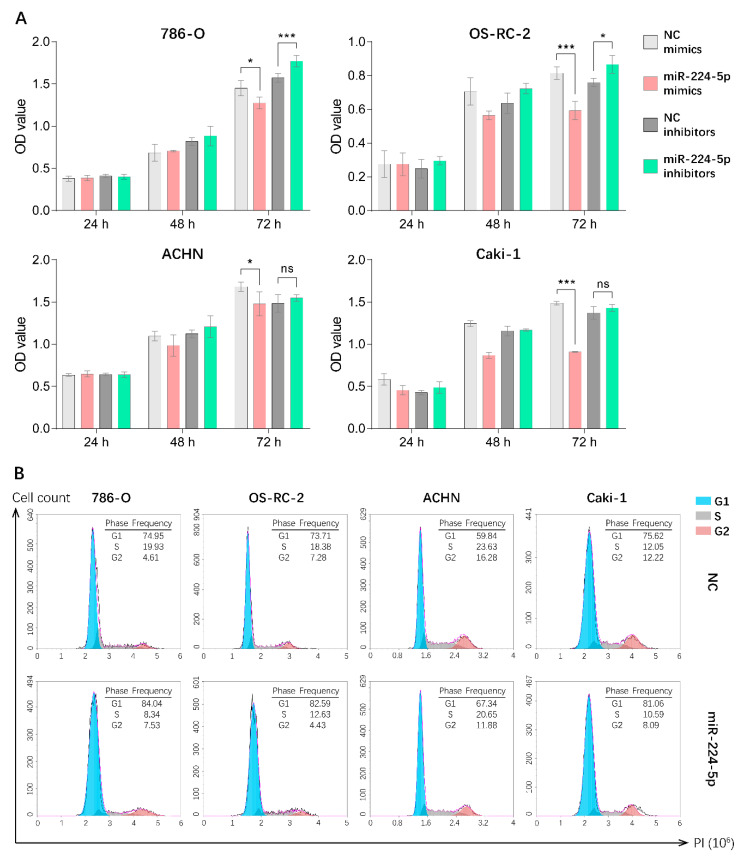
miR-224-5p inhibited RCC cell proliferation and induced cell cycle arrest. (**A**) Cell proliferation of miR-224-5p mimic/inhibitor-transfected RCC cells was detected by CCK-8 assay. Data are mean ± s.e.m. * *p* < 0.05; *** *p* < 0.001; ns, no significant difference. (**B**) Cell cycle distributions after transfection of miR-224-5p mimics into RCC cells were determined by flow cytometry. The frequency of each cell cycle phase is listed in figure.

Since miR-224-5p was overexpressed in urinary EVs and cancer tissues of RCC patients, it is reasonable to unveil the detailed roles of miR-224-5p in RCC progression. Hence, CCK-8 assays were performed in order to investigate the potential effect of miR-224-5p on RCC cell proliferation. Results indicated that the overexpression of miR-224-5p by mimics significantly inhibited the proliferation of 786-O, OS-RC-2, ACHN and Caki-1 cells. Transfection of miR-224-5p inhibitors in 786-O and OS-RC-2 markedly reversed this inhibitory effect compared to NC inhibitors, but no significant differences were presented in ACHN and Caki-1 cells (Figure 4A). Additionally, flow cytometry was performed to investigate whether miR-224-5p is involved in the regulation of cell cycle status in RCC cells. Results suggested that an increase in the percentage of cells in the G1 phase and a decrease in the percentage of S-phase cells were present in miR-224-5p mimic-transfected RCC cells, respectively (Figure 4B). In addition, the percentage of cells in the G1 phase decreased after RCC cells were transfected by miR-224-5p inhibitors (Figure S3). This trend was not significant, which may be due to the relatively low levels of miR-224-5p expression in RCC cells (RT-qPCR Ct values > 30). The above results suggested that miR-224-5p can inhibit the cell proliferation and induce cell cycle arrest in RCC.

### 2.5. miR-224-5p Regulated Cyclin D1 Expression in RCC Cells

**Figure 5:**
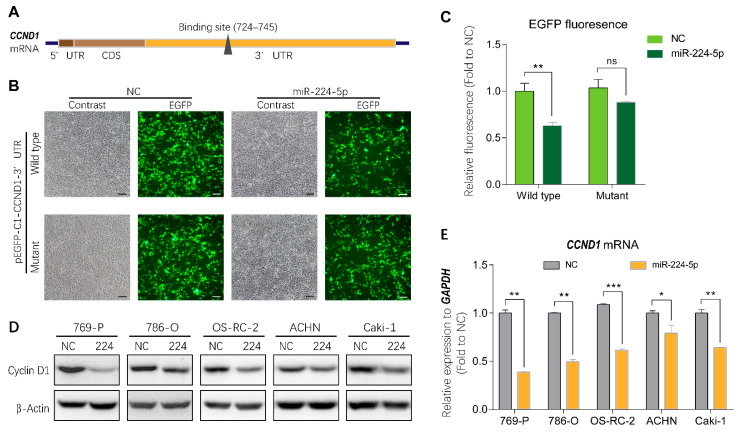
Cyclin D1 expression was regulated by miR-224-5p. (**A**) Schematic of potential binding sites of miR-224-5p in the 3′UTR region of *CCND1* mRNA. (**B**,**C**) GFP fluorescence in HEK293 cells after 48 h of co-transfection with indicated miRNA mimics (NC or miR-224-5p) and pEGFP-C1-*CCND1*-3′UTR wildtype/mutant plasmids. Scale bar, 100 nm. Fluorescent value of GFP (relative fluorescence) was normalized to concentration of total protein and shown as the fold of NC group. Data are mean ± s.e.m. ** *p* < 0.01; ns, no significant difference. (**D**) The regulation of cyclin D1 protein in RCC cells by miR-224-5p was determined by Western blot. (**E**) The regulation of *CCND1* mRNA in RCC cells by miR-224-5p was determined by RT-qPCR. Data are mean ± s.e.m. * *p* < 0.05; ** *p* < 0.01; *** *p* < 0.001. (Original Western blots see Figure S7).

To investigate the mechanisms of miR-224-5p-induced cell cycle arrest in RCC, bioinformatics tools were applied to identify direct target genes of miR-224-5p. As shown in Figure 5A, cyclin D1 mRNA (*CCND1*) was predicted using the miRTarBase and miRWalk databases as a putative target of miR-224-5p containing a potential binding site in the 3′UTR region. Thus, the wild type and mutant of the putative binding site were cloned into the pEGFP-C1 vector, followed by a stop codon to terminate the translation of enhanced green fluorescent protein (EGFP). Results of fluorescent reporter gene assays suggested that EGFP fluorescence in the miR-224-5p group was significantly decreased compared to that of the NC group co-transfected with wild type pEGFP-C1-*CCND1*-3′UTR plasmid in HEK-293 cells. Meanwhile, there was no significant difference between miR-224-5p and NC mimics co-transfected with the mutant plasmid (Figure 5B,C). These results demonstrated that miR-224-5p downregulated *CCND1* expression by targeting its binding site in the 3′UTR region. More importantly, miR-224-5p mimics inhibited the expression of cyclin D1 protein in RCC cells as shown in Figure 5D. In addition, *CCND1* mRNA levels in RCC cells were significantly lower after transfection with miR-224-5p mimics than in NC groups (Figure 5E). Therefore, it can be concluded that miR-224-5p may downregulate cyclin D1 expression in RCC cells.

### 2.6. miR-224-5p Upregulated PD-L1 Expression through the Cyclin D1/SPOP Pathway

**Figure 6:**
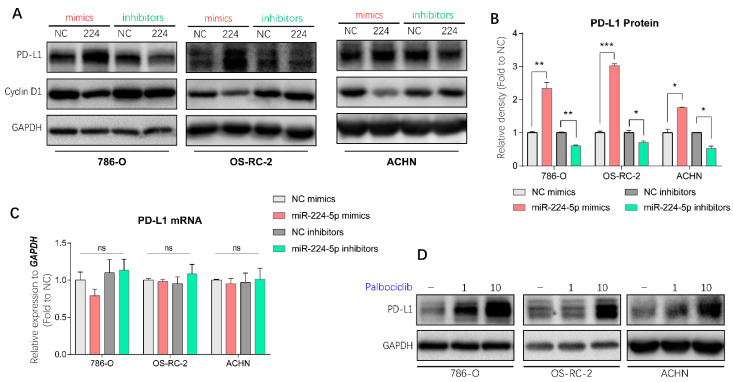
miR-224-5p involved in the regulation of PD-L1 protein in RCC cells by inhibiting cyclin D1 expression. (**A**,**B**) PD-L1 protein abundances in RCC cells were detected by Western blot after transfection of miRNA mimics/inhibitors. The relative density of PD-L1 protein is shown as mean ± s.e.m of triplicate experiments. * *p* < 0.05; ** *p* < 0.01; *** *p* < 0.001. (**C**) PD-L1 mRNA levels in RCC cells were determined by RT-qPCR after transfection of miRNA mimics/inhibitors (100 nM). Data are mean ± s.e.m. ns, no significant difference. (**D**) PD-L1 protein abundances in RCC cells were detected by Western blot after treatment of selective CDK4/6 inhibitor palbociclib (1 or 10 μM) for 48 h. (Original Western blots see Figure S7).

It has been demonstrated in a previous study that PD-L1 protein stability is regulated by cyclin-D-CDK4 and cullin 3-SPOP E3 ligase via proteasome-mediated degradation [7]. Since miR-224-5p interrupted cyclin D1 expression in RCC cells, it is reasonable to infer that miR-224-5p may also be involved in the regulation of PD-L1 expression. PD-L1 mRNA and protein abundances were determined in paired cancer and adjacent tissues of 12 RCC patients, of which eight specimens were inconsistent at the mRNA and protein levels (Figure S4). Hence, there may be unclear post-translational mechanisms involved in PD-L1 protein expression in RCC. Interestingly, results of Western blot analysis indicated that miR-224-5p mimics promoted PD-L1 protein levels in 786-O, OS-RC-2 and ACHN cells, and this induction could be reversed by miR-224-5p inhibitors (Figure 6A,B). Since the original mRNA levels of *CCND1* in these RCC cell lines (mean Ct values of RT-qPCR range from 19.7 to 21.5) were much higher than the levels of miR-224-5p (RT-qPCR Ct values > 30), it can be inferred that the effects of miR-224-5p inhibitors on cyclin D1 protein expression were relatively weak. Meanwhile, there was no effects of miR-224-5p mimics or inhibitors on PD-L1 mRNA levels (Figure 6C). Similarly, PD-L1 protein expressions were increased by small interfering RNAs (siRNA) targeting *CCND1* (Figure S5A), or by treatment of the selective CDK4/6 inhibitor palbociclib (Figure 6D). Cullin-based ubiquitin E3 ligase inhibitor MLN4924 and proteasome inhibitor MG132 also upregulated PD-L1 protein levels in 786-O, OS-RC-2 and ACHN cells (Figure S5B). Collectively, these results demonstrated that cyclin D-CDK4/6 also plays a rate-limiting role in regulating PD-L1 expression in RCC cells. miR-224-5p may be involved in the regulation of PD-L1 protein stability by inhibiting cyclin D1 expression, followed by interruption of cyclin D-CDK4/6 and cullin 3-SPOP E3 ligase-mediated proteasome degradation pathways.

### 2.7. EVs Transmitted the Regulation of miR-224-5p in PD-L1 Expression

**Figure 7:**
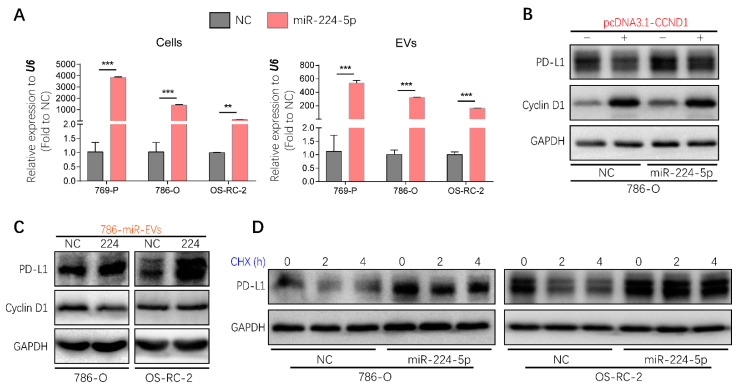
Regulation of PD-L1 protein expression by miR-224-5p inhibiting cyclin D1 expression could be transmitted between RCC cells by EVs. (**A**) miR-224-5p levels in RCC-NC/miR-224-5p cells and EVs were determined by RT-qPCR (** *p* < 0.01; *** *p* < 0.001). (**B**) Protein levels of cyclin D1 and PD-L1 in *CCND1* transiently expressed 786-O-NC/miR-224-5p cells were detected by Western blot. (**C**) Protein levels of PD-L1 and cyclin D1 in RCC cells co-culturing with EVs derived from 786-O-NC/miR-224-5p cells for 24 h were detected by Western blot. (**D**) PD-L1 protein stability in RCC-NC/miR-224-5p cells was detected after CHX (25 mg/mL) treatment for indicated time (h) as shown in the figure. (Original Western blots see Figure S7).

Considering that EVs represent one of the mediators of cell–cell communication, it will be valuable to investigate whether the regulation of miR-224-5p on PD-L1 levels through inhibiting cyclin D1 could be transmitted between RCC cells via EVs. Hence, miR-224-5p stably overexpressed RCC cells (RCC-miR-224-5p) were produced using a lentivirus system; meanwhile, negative control cells (RCC-NC) were constructed by a cloned non-sense sequence. Then EV-containing miRNAs were extracted from RCC-NC/miR-224-5p cells. Results showed that miR-224-5p levels were significantly higher in EVs derived from RCC-miR-224-5p cells than in RCC-NC cells, which were consistent with the levels in parent cells (Figure 7A). Furthermore, the trends of cyclin D1 and PD-L1 protein abundance in RCC-NC/miR-224-5p cells were similar to that in miRNA mimic-transfected RCC cells (Figure S6A), which could be reversed by miR-224-5p inhibitors (Figure S6B), or the transient overexpression of *CCND1* (Figure 7B).

EVs derived from 786-O-NC/miR-224-5p cells were labeled with PKH67 and co-cultured with RCC cells to verify the uptake process. As shown in Figure S6C, green fluorescence of PKH67 was observed in intracellular areas. After co-culture with 786-O-NC/miR-224-5p cell-derived EVs, a decreased abundance of cyclin D1 was present in 786-O cells but not in OS-RC-2 cells, which may be due to the higher level of miR-224-5p in OS-RC-2 cells than that in 786-O cells. However, both these two RCC cells exhibited increased levels of PD-L1 protein (Figure 7C). Given that miR-224-5p may induce PD-L1 abundance by improving protein stability, the cycloheximide (CHX)-chase assay was conducted to inhibit de novo protein synthesis. The results indicated that the degradation process of PD-L1 protein was inhibited in RCC-miR-224-5p cells compared to RCC-NC cells (Figure 7D). Hence, it can be inferred that RCC-miR-224-5p cells could be a donor to release EVs containing miR-224-5p and deliver cargoes to recipient cells deferring PD-L1 protein degradation by inhibiting cyclin D1 expression.

### 2.8. miR-224-5p Enhanced Resistance to T Cell-Dependent Toxicity, Invasive and Metastatic Abilities of RCC Cells

**Figure 8:**
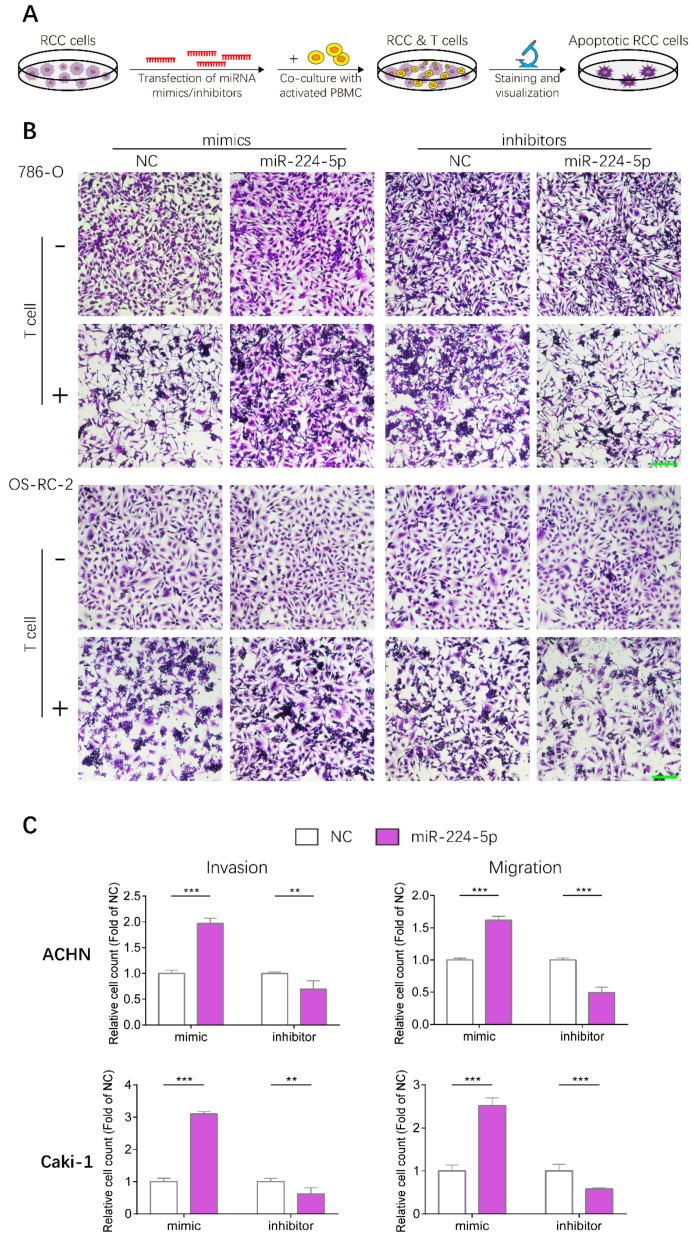
miR-224-5p enhanced resistance to T cell-dependent toxicity, invasive and metastatic abilities of RCC cells. (**A**) Schematic of T cell-dependent RCC cells killing assay. 786-O and OS-RC-2 cells were transfected by miRNA mimcs/inhibitors (100 nM) for 48 h. T cells were isolated from fresh whole blood of healthy volunteers and activated by anti-CD3/CD28/CD2 antibodies for 3 days, and co-cultured with RCC cell for 24 h. Viable RCC cells were stained by crystal violet and visualized by a microscope. (**B**) Representative images of stained viable RCC cells. Scale bar, 200 μm. (**C**) miR-224-5p enhanced invasive and metastatic abilities of RCC cells. Representative images of cell invasion and migration after transfection of miR-224-5p mimics/inhibitors into ACHN and Caki-1 cells were obtained by an inverted microscope. Cell numbers were counted using Image J software from 5 random fields. Data are means ± s.e.m. ** *p* < 0.01; *** *p* < 0.001.

Given that PD-L1 levels in RCC cells could be upregulated by miR-224-5p through inhibition of cyclin D1 expression, whether this mechanism could render RCC cells resistant to T cell-mediated toxicity was further investigated by conducting T cell killing assays, as shown in Figure 8A. Human peripheral blood mononuclear cells (PBMC) were isolated from fresh whole blood of healthy volunteers, and T cells were activated then co-incubated with 786-O and OS-RC-2 cells transfected with miR-224-5p or NC mimics/inhibitors. Viable RCC cells were stained and visualized. The results suggested that more RCC cells were alive after transfection of miR-224-5p mimics compared to NC, and this effect could be reversed by miR-224-5p inhibitors (Figure 8B). Thus, it can be inferred that miR-224-5p promoted RCC cell resistance to T cell-dependent toxicity. Moreover, the effects of miR-224-5p on the invasive and metastatic abilities of RCC cells were also investigated. ACHN and Caki-1, two RCC cell lines derived from metastatic sites, were chosen for transfection of miRNA mimics/inhibitors and then seeded into Transwell^TM^ inserts. As shown in Figure 8C, the invasive and metastatic abilities of ACHN and Caki-1 cells overexpressing miR-224-5p were both significantly enhanced compared to the NC groups. On the contrary, miR-224-5p inhibitors significantly suppressed the invasion and migration of ACHN and Caki-1 cells compared to NC groups. Therefore, it can be concluded that miR-224-5p may promote invasion and migration of RCC cells.

## 3. Discussion

Recently, various isolation and characterization techniques have been developed in the burgeoning field of EVs [8]. In this study, urinary EVs from RCC patients and healthy volunteers were isolated by differential UC, since purity of EVs prepared from this method is much higher than with commercial isolation kits [14]. Typical techniques that are recommended by the International Society for Extracellular Vesicles were utilized to characterize EVs [15]. Small RNA sequencing was then implemented to profile urinary EVs containing miRNAs derived from RCC patients and healthy volunteers. Considering expression levels and patterns, three upregulated miRNAs (miR-1-3p, miR-150-5p and miR-224-5p) that had been annotated previously were screened to verify their expression levels in RCC tissues [16,17]. Results showed that only the levels of miR-224-5p were consistent with data from tissue specimens and the TCGA database, which has also been described in previous studies [18,19,20]. Therefore, detection of miR-224-5p levels in EVs derived from bodily fluids will contribute to the development of miR-224-5p as a non-invasive biomarker for RCC diagnosis, prognosis and treatment. However, to date, only one study has determined the level of miR-224 in serum-derived EVs, which has been described as a significant risk factor in RCC prognosis [21]. To the best of our knowledge, no study has focused on miR-224-5p levels in urinary EVs of RCC patients. Hence, the isolation and characterization methods employed in this study will provide new insight into the detection of miR-224-5p in urinary EVs, and lay groundwork for the application of miR-224-5p as a biomarker in RCC.

Based on the significant upregulation of miR-224-5p in both urinary EVs and tissue specimens of RCC samples, we were encouraged to investigate its biological functions by in vitro studies. It has been reported that an inverse correlation exists between the expression of miR-224-5p and iodothyronine deiodinase 1 in tissues of clear cell RCC patients [22]. Similarly, Lichner et al. revealed that miR-224-5p expression in RCC specimens is negatively correlated with SMAD4/5, which are key regulators downstream of the TGF-β pathway [23]. Moreover, Liao et al. reported that miR-224-5p directly targets the tumor suppressors PH domain leucine-rich-repeats protein phosphatase 1 and 2 in colorectal cancer cells. Their study indicated that miR-224-5p promotes cell proliferation, accelerates cell cycle G1-S phase transition and upregulates cyclin D1 expression [24]. Zhang et al. reported that miR-224-5p suppresses apoptosis and activates proliferation of gastric cancer cells by modulating caspase-9/3 and cyclin D1/2, although there were no significant differences in cyclin D1/2 expression after the overexpression of miR-224-5p [25]. However, studies also shown that miR-224-5p acts as a tumor suppressor in uveal melanoma cells. The ectopic expression of miR-224-5p attenuates cell proliferation by targeting lncRNA FTH1P3 or PIK3R3/AKT3 pathway [26,27]. In addition, a recent study revealed that LINC01094, a long noncoding RNA that is upregulated in clear cell RCC, works as a sponge of miR-224-5p and exerts tumor-promoting effects in RCC progression [28]. These findings suggest that miR-224-5p orchestrates multiple processes in the development of various cancer cells. Hence, certain upstream regulators may be involved in the expression of miR-224-5p, which may influence the downstream effects of miR-224-5p on RCC progression. Interestingly, our study revealed that miR-224-5p interrupted the processes of RCC cell proliferation and cell cycle by inhibiting cyclin D1 expression. The metastatic and invasive abilities as well as the resistance to T cell-mediated toxicity of RCC cells were all enhanced. Therefore, it can be inferred that miR-224-5p may be upregulated in the advanced stage of RCC and play important roles in the processes of metastasis and immune suppression of RCC cells. In summary, detailed roles of miR-224-5p in cancer progression are still controversial and remain to be further clarified.

Previous study has revealed that pharmacological inhibitors of CDK4/6 not only induce tumor cell cycle arrest but also promote anti-tumor immunity [29]. Moreover, PD-L1 abundance fluctuates during cell cycle progression in various cancer cell lines, which is regulated by cyclin D-CDK4 and cullin 3-SPOP E3 ligase via proteasome-mediated degradation [7]. However, the mechanistic pathways of PD-L1 protein expression in RCC cells and the role of miR-224-5p in immune evasion remain unknown. In this study, we demonstrated that miR-224-5p is involved in the regulation of PD-L1 protein stability in RCC cells through inhibiting cyclin D1 expression. Interestingly, this regulatory mechanism could be transmitted between RCC cells via EVs. Therefore, miR-224-5p carried by urinary EVs has the potential to be a predictive biomarker for PD-1/PD-L1 blockade therapy in RCC patients. Furthermore, Haderk et al. revealed that non-coding Y RNA hY4 is enriched in EVs from the plasma of chronic lymphocytic leukemia patients. PD-L1 expression in monocytes could be induced by treatment of these EVs or hY4 alone [30]. It is valuable to investigate whether these interesting effects exist in RCC patient-derived EVs. Emerging studies also demonstrated the presence of PD-L1 protein with full activity in EVs from various cancer cells, including glioblastoma, melanoma and prostate cancer cells [31,32,33,34,35]. Thus, to understand the detailed roles of PD-L1 in immune evasion processes of RCC cells, it is necessary to detect the PD-L1 abundance at both parental cell and EV levels. Inhibition of EV biogenesis through genetic or pharmacological approaches may make a contribution to blockading the function of EV-packaged PD-L1. Recently, Incorvaia et al. found that the expression of several miRNAs derived from peripheral lymphocytes of RCC patients was associated with the soluble levels of PD-1 and PD-L1 in plasma. These miRNA predictors could be used to discriminate responders of immunotherapy from non-responders [36]. This work also provides reliable clues to clarify the association of miRNA expression profile and PD-1/PD-L1 abundances in various EVs of RCC patients. Altogether, further investigations need to be implemented to discover novel biomarkers for the prediction of potential response in RCC immunotherapy. 

## 4. Materials and Methods

### 4.1. Cell Culture and Treatment of Chemical Inhibitors

The HEK-293 cell line and three RCC cell lines (769-P, 786-O and Caki-1) were purchased from the Cell Bank of Committee on Type Culture Collection of the Chinese Academy of Sciences (Shanghai, China). RCC cell line OS-RC-2 was obtained from the Medical College of Zhejiang University as a gift from Professor Maode Lai in 2017. RCC cell line ACHN was revived from our laboratory. HEK-293 cells were cultured in DMEM medium (10-013-CVR, Corning, New York, NY, USA) supplemented with 10% fetal bovine serum (FBS; 10099-141, Gibco, Gaithersburg, MD, USA) and 1% penicillin/streptomycin (P1400, Solarbio, Beijing, China). 769-P and 786-O cells were maintained in RPMI 1640 medium (C11875500BT, Gibco, USA) supplemented with 10% FBS, 1% penicillin/streptomycin and 1% sodium pyruvate (S8636, Sigma-Aldrich, St. Louis, MO, USA). OS-RC-2 cells were cultured in RPMI 1640 medium supplemented with 10% FBS and 1% penicillin/streptomycin. ACHN cells were cultured in MEM medium (10-010-CVR, Corning, USA) supplemented with 10% FBS and 1% penicillin/streptomycin. Caki-1 cells were cultured in McCoy’s 5A medium (GNM16600, Genom, Hangzhou, China) supplemented with 10% FBS, 1% penicillin/streptomycin and 1% sodium pyruvate. All cells were maintained in a 5% CO2 incubator at 37 °C with saturated humidity and routinely tested for mycoplasma contamination by a MycoGuard^TM^ Mycoplasma PCR detection kit (MP001, GeneCopoeia, Rockville, MD, USA) according to the manufacturer’s protocol.

The CDK4/6 inhibitor palbociclib (T1785, TargetMol, Boston, MA, USA), cullin-based ubiquitin E3 ligase inhibitor MLN4924 (T6332, TargetMol, USA), proteasome inhibitor MG132 (S2619, SelleckChem, Houston, TX, USA) and protein synthesis inhibitor cycloheximide (CHX, A10036, Adooq, Irvine, CA, USA) were all dissolved in DMSO to prepare store solutions in necessary concentrations. RCC cells were seeded into 12-wells plate and allowed to reach 30 to 75% confluence prior to chemical treatment. The next day, RCC cells were exposed to completed culture medium containing chemical inhibitors to reach a 0.1% volume fraction. The control group was treated with 0.1% DMSO. After a further incubation, total protein of RCC cells was extracted for further use. The final concentration and incubation period of each chemical inhibitor are indicated in the results section.

### 4.2. Clinical Specimen Collection

Paired cancer and adjacent tissues of 35 RCC patients, urine samples of 6 RCC patients and 6 healthy volunteers were collected from Zhejiang Cancer Hospital (Hangzhou, China) after signed informed consents. All tissue specimens were collected after surgical treatment and frozen in liquid nitrogen immediately, then stored at −80 °C in the Specimen Bank of Zhejiang Cancer Hospital for further analysis. Urine samples of RCC patients were collected prior to surgery and delivered to the laboratory on ice, and then processed to isolate EVs promptly or stored at −80 °C for further use. The study was scrutinized and approved by the Ethics Committee of the Zhejiang Cancer Hospital (IRB-2017-02). Detailed specimen information is summarized in supplemental the information (Tables S1 and S2).

### 4.3. Isolation, Characterization and Fluorescence Staining of EVs

EVs derived from the culture medium of RCC cells were isolated as described in our previous study [13]. Human urine-derived EVs were isolated by differential UC as previously described and with minor modifications [37]. First, urine samples with volumes ranging from 50 to 240 mL were centrifuged at 17,000× *g* for 15 min at 4 °C. The supernatant was collected and filtered through a 0.22 μm pore filter (SLGP033RS, Millipore, Burlington, MA, USA). Next, the filtrate was ultracentrifuged at 150,000× *g* for 2 h at 4 °C. EV pellets were resuspended in PBS buffer, followed by a repeated UC at 150,000× *g* for 2 h at 4 °C to pellet EVs. Finally, EV pellets were resuspended in different reagents according to the needs of subsequent studies. To characterize the shape and size distribution of EVs, pellets were resuspended in PBS buffer and examined by TEM (Tecnai 10, Philips, Eindhoven, The Netherlands) and NTA (ZetaView^TM^, Particle Metrix, Inning am Ammersee, Germany), respectively. RIPA lysis buffer (P0013B, Beyotime, Shanghai, China) was utilized to detect EV-specific proteins by Western blot analysis. To extract EV-containing RNA, EV pellets were lysed using QIAzol^TM^ lysis reagent (79306, Qiagen, Hilden, Germany) and processed as per the instructions of the miRNeasy^TM^ Micro kit (217084, Qiagen, Germany).

To observe the process of EV uptake by recipient cells, EVs derived from RCC cell culture medium were labeled with a PKH67 Green Fluorescence Cell Linker kit (MINI67-1KT, Sigma-Aldrich, USA) according to the manufacturer’s protocol. Recipient cells were co-cultured with PKH67-labeled EVs for 24 h. Cell membrane and nuclei were stained with Dil (C1036, Beyotime, China) and DAPI (C1005, Beyotime, China), respectively. The stained EVs and recipient cells were visualized using a high-resolution confocal laser-scanning microscope (LSM 880 with AiryScan, Zeiss, Oberkochen, Germany).

### 4.4. RNA Isolation and Real-Time Quantitative PCR

According to the manufacturer’s instructions, total RNA derived from tissues, cell lines and EVs was isolated by an RNA Simple^TM^ RNA kit (DP419, Tiangen, Beijing, China), AxyPrep^TM^ RNA Miniprep kit (AP-MN-MS-RNA-250, Axygen, Union City, CA, USA) and miRNeasy^TM^ Micro kit, respectively. Then, the RNA sample was reverse transcribed to cDNA with random primers using a PrimeScript^TM^ RT Master Mix kit (RR036A, Takara, Shiga, Japan). Specific stem-loop primers (Table 1) were used to detect miRNAs by an miRNA 1st Strand cDNA Synthesis kit (MR101-02, Vazyme, Nanjing, China). RT-qPCR was performed by using a Real-Time PCR System (StepOnePlus, Applied Biosystems, Waltham, MA, USA) with TB Green Premix Ex Taq^TM^ (RR420A, Takara, Japan), as described in our previous study [38]. RT-qPCR-related primers are listed in Table 2. The relative mRNA expression level of each target gene was normalized to GAPDH or RNU6-1 (U6).

### 4.5. miRNA Library Construction and Sequencing

EV-containing RNA derived from urine samples of healthy volunteers and RCC patients was used for small RNA library preparation and sequencing, which were performed at Novogene (Tianjin, China). Briefly, the amount and size distribution of EV-containing total RNA were determined using an Agilent 2100 pic600 Bioanalyzer instrument. The cDNA libraries of small RNA within total RNA samples were obtained by reverse transcription, PCR amplification, gel purification and size selection. Next, the libraries were qualified using the Agilent 2100 Bioanalyzer system and sequenced by the Illumina Hiseq^TM^ SE500 platform. Differential expression analysis of the two groups was performed using DEseq2 [39]. The default threshold for statistically differential expression was set as *p* values < 0.05.

### 4.6. Western Blot Analysis

Proteins were extracted from EVs or cells using RIPA lysis buffer supplemented with protease inhibitor PMSF (ST506, Beyotime, China) and measured by BCA protein assay kit (P0011, Beyotime, China) to normalized protein concentrations. Equal amounts of protein were fractioned using SDS-PAGE by 5% stacking gels and 10% separation gels, and transferred to polyvinylidene difluoride membrane (IPVH00010, Millipore, USA). Membranes were blocked by 5% non-fat milk for 2 h at room temperature, then immunoblotted with indicated primary antibodies overnight at 4 °C, including anti-GRP94 (1:5000, ab3674, Abcam, Bristol, UK), anti-ALIX (1:2000, ab117600, Abcam, UK), anti-TSG101 (1:500, sc-7964, Santa Cruz, Santa Cruz, CA, USA), anti-CD63 (1:2000, ab59479, Abcam, UK), anti-β-Actin (1:5000, ab40009, MiltiSciences, Hangzhou, China), anti-Cyclin D1 (1:2000, ab16663, Abcam, UK), anti-GAPDH (1:5000. 60004-1-Ig, Proteintech, Rosemont, IL, USA) and anti-PD-L1 (1:1000, 13684, Cell Signaling Technology, Danvers, MA, USA). Horseradish peroxidase-conjugated goat anti-mouse IgG (H+L) (1:5000, 70-GAM0072, Multi Sciences, China) or goat anti-Rabbit IgG (H+L) (1:5000, 70-GAR0072, Multi Sciences, China) was utilized as secondary antibody to incubate with membranes at room temperature for 1 h. Blots were visualized by applying the G-BOX gel imaging system (Chemi XR 5, Syngene, Cambridge, UK).

### 4.7. Plasmid and Transfection

Putative targets of miR-224-5p were predicted and evaluated using bioinformatics tools, including miRTarBase and miRWalk databases [40,41]. *CCND1* was chosen as the target gene in this study. The full length of the 3′ untranslated region (3′UTR) of *CCND1* gene is 4238 bp. A 695 bp region (from 108 to 802 bp) of the *CCND1* 3′UTR containing a potential miR-224-5p binding site was cloned into the pEGFP-C1 plasmid vector. Moreover, a stop codon was inserted between the EGFP coding sequence and multiple cloning sites to terminate EGFP translation. Construction of multiple pEGFP-C1 vectors with wild type and mutant miR-224-5p binding sites in the *CCND1* 3′UTR (pEGFP-C1-*CCND1*-3′UTR) was conducted by Tsingke Biological Technology (Hangzhou, China). miR-224-5p mimics or inhibitors, NC miRNA and siRNA targeting *CCND1* were purchased from GenePharma (Shanghai, China). Lipofectamine 2000 reagent (11668-019, Invitrogen, Waltham, MA, USA) was used to transfect plasmid, miRNA or siRNA into RCC cells following recommended protocols.

### 4.8. Fluorescence Reporter Assay

HEK-293 cells were seeded into 24-well plates and allowed to reach 50% confluence prior to transfection. Equal amounts of miR-224-5p or NC mimics (100 nM) were co-transfected with wildtype or mutant pEGFP-C1-*CCND1*-3′UTR plasmids (100 ng/well) using Lipofectamine 2000 reagent. After 48 h of transfection, EGFP fluorescent intensities of EGFP (488-518 nm) were determined using a Synergy^TM^ H1 multifunctional microplate reader (BioTek, Winooski, VT, USA). EGFP fluorescence was also visualized using an inverted fluorescent microscope (Eclipse Ti-S, Nikon, Tokyo, Japan). Total protein content of each sample was harvested using RIPA lysis buffer and protein concentration was detected using the BCA assay. The EGFP value of each sample was normalized to the total protein concentration.

### 4.9. Construction of miR-224-5p Stable Expressed Cell Lines

A lentivirus system was purchased from Genechem (Shanghai, China) to construct miR-224-5p stably overexpressed RCC cells (RCC-miR-224-5p). Meanwhile, a non-sense sequence (TTCTCCGAACGTGTCACGT) was cloned as a negative control (RCC-NC). Elements including hU6-MCS-Ubiquitin-EGFP-IRES-puromycin were sequentially added to the lentivirus expression vector. After infection of RCC cells, stably expressing RCC cells were selected by puromycin (A1113803, Gibco, USA). Fluorescence of green fluorescent protein (GFP) was observed and recorded using an inverted fluorescent microscope. The expression level of miR-224-5p in each stable-expressed cell line was determined by quantitative RT-qPCR.

### 4.10. Cell Proliferation Assays and Cell Cycle Analysis

For cell proliferation assays, RCC cells were seeded into 96-well plates prior to transfection with the cell count ranging from 2500 to 5000 cells/well for different cell lines according to their proliferation rate. Then miR-224-5p or NC mimics/inhibitors with a final concentration of 100 nM were transfected into RCC cells by Lipofectamine 2000 reagent. After a series of gradient incubation periods ranging from 24 to 72 h at 37 °C with 5% CO_2_, CCK-8 solution (c6030, NCM Biotech, Suzhou, China) was added to each well in fresh medium and incubated for an additional 1 h. The absorbance was measured at 450 and 650 nm using a multifunctional microplate reader.

For cell cycle analysis, RCC cells were seeded into 6-well plates to reach a confluence of 50% in prior to transfection. RCC cells were transfected by miR-224-5p or NC mimics/inhibitors with a final concentration of 100 nM using Lipofectamine 2000. After an additional 48 h of incubation, RCC cells were harvested by trypsin, fixed by 70% ethanol and then stained by propidium (PI) following the manufacturer’s instructions for the cell cycle assay kit (C1052, Beyotime, China). PI-stained samples were detected by a NovoCyte^TM^ flow cytometer (ACEA Biosciences, San Diego, CA, USA). Results were analyzed by NovoExpress^TM^ software (ACEA Biosciences, San Diego, CA, USA).

### 4.11. PBMC Isolation and Co-Culture of RCC Cells with T Cells

To acquire PBMC, fresh whole blood samples of healthy volunteers were obtained from a hospital, and then isolated by Ficoll-Paque PLUS density gradient media (17144002; Cytiva, Marlborough, MA, USA) according to the manufacturer’s protocol. The isolated PBMC were cultured in TexMACS Medium (130-097-196; Miltenyi, Bergisch Gladbach, Germany) with 10 ng/mL recombinant human IL-2 (200-02; PeproTech, Cranbury, NJ, USA). T cells were activated by adding 25 μL/mL ImmunoCult™ Human CD3/CD28/CD2 T Cell Activator (10970; Stemcell, Seattle, WA, USA) for 3 d. During this period, RCC cells were seeded and transfected with miR-224-5p or NC mimics/inhibitors for 48 h before co-culture with activated T cells. The ratio between RCC and T cells in the co-culture was 1:5. T cells and cellular debris were removed by washing with PBS buffer after 24 h of co-culture, and then living cancer cells were stained using crystal violet.

### 4.12. Cell Migration and Invasion Assays

RCC cells were transfected with miR-224-5p or NC mimics/inhibitors with a final concentration of 100 nM using Lipofectamine 2000 for 48 h. After this period, RCC cells were harvested and washed by PBS buffer 2 times, then resuspended in FBS-free culture medium with a final amount of 2 × 10^5^ cells/well (ACHN cells) or 1 × 10^5^ cells/well (Caki-1 cells). Then, RCC cells were seeded into Transwell^TM^ inserts (3422, Corning, USA) with or without Matrigel^TM^ basement membrane matrix (356234, Corning, USA) for invasion and migration assay, respectively. The lower chamber was filled with 650 μL completed culture medium containing FBS. After a 24 h continuous incubation, cells adhering to the bottom of inserts were fixed by 4% paraformaldehyde and stained by crystal violet solution. Then, stained cells were visualized using an inverted fluorescent microscope and counted by image processing software (Image J, NIH, Bethesda, MD, USA) automatically. 

### 4.13. Statistical Analysis

Data are representative of at least three independent experiments, and are expressed as means ± s.e.m. Differences among groups were analyzed using a Student’s *t*-test, and *p* < 0.05 was considered statistically significant.

## 5. Conclusions

In summary, miR-224-5p contained in urinary EVs was found to be significantly upregulated in RCC patients compared to healthy volunteers. Moreover, the inhibition of miR-224-5p on cyclin D1 expression may elucidate new dimensions of miR-224-5p biological functions in RCC progression. The inductive mechanisms of PD-L1 protein abundance in RCC cells by miR-224-5p may provide a molecular basis for the development of miR-224-5p as a promising non-invasive biomarker for immunotherapies in RCC treatment.

## Figures and Tables

**Table 1:** Stem-loop primers of miRNAs.

Gene	Stem-Loop Primer (5′ to 3′)
*RNU6-1 (U6)*	GTCGTATCCATGGCAGGGTCCGAGGTATTCGCCATGGATACG
*miR-1-3p*	GTCGTATCCAGTGCAGGGTCCGAGGTATTCGCACTGGATACGACATACAT
*miR-150-5p*	GTCGTATCCAGTGCGTGTCGTGGAGTCGGCAATTGCACTGGATACGACCACTGG
*miR-224-5p*	GTCGTATCCAGTGCGTGTCGTGGAGTCGGCAATTGCACTGGATACGACCTAAAC

**Table 2:** RT-qPCR primers.

Gene	Forward (5′ to 3′)	Reverse (5′ to 3′)
*U6*	CTCGCTTCGGCAGCACA	AACGCTTCACGAATTTGCGT
*miR-1-3p*	CGCGCTGGAATGTAAAGAAGT	GTGCAGGGTCCGAGGT
*miR-150-5p*	GGGTCTCCCAACCCTTGTA	CAGTGCGTGTCGTGGAGT
*miR-224-5p*	GCTCAAGTCACTAGTGGTTCC	CAGTGCGTGTCGTGGAGT
*GAPDH*	AGGTGAAGGTCGGAGTCA	GGTCATTGATGGCAACAA
*CCND1*	TGAACTACCTGGACCGCT	GCCTCTGGCATTTTGGAG
*PD-L1*	TGGCATTTGCTGAACGCATTT	TGCAGCCAGGTCTAATTGTTTT

## Data Availability

The data presented in this study are available on request from the corresponding author.

## Supplementary Materials

The following are available online at https://www.mdpi.com/2072-6694/13/4/618/s1, Figure S1: Heatmap of the 61 miRNAs differentially expressed in urinary EVs of RCC patients and healthy volunteers (*p* < 0.05). Figure S2: Expression levels of miR-1-3p and miR-150-5p in tissues of RCC patients. (A) Expression levels of miR-1-3p and miR-150-5p in cancer and adjacent tissues of RCC samples used in small RNA-sequencing were determined by RT-qPCR. (B) Expression levels of miR-1-3p and miR-150-5p in paired tissues of RCC patients were determined by RT-qPCR (n = 30). * *p* < 0.05; ** *p* < 0.01; *** *p* < 0.001; ns, no significant difference. Figure S3: Cell cycle distributions after transfection of miR-224-5p inhibitors into RCC cells were determined by flow cytometry. Figure S4: PD-L1 mRNA (A) and protein (B) expression levels of in paired adjacent and cancer tissues of 12 RCC patients were determined by RT-qPCR and Western blot, respectively. Figure S5: Regulation of PD-L1 protein expression by cyclin D-CDK4/6 and cullin 3-SPOP E3 ligase mediated proteasome degradation. (A) *CCND1* mRNA levels in RCC cells were determined by RT-qPCR after transfection of siRNAs targeting *CCND1* (siR-1, siR-2, and siR-3). (B) PD-L1 protein abundance in RCC cells was detected by Western blot after transfection of siRNAs targeting *CCND1*. (C) PD-L1 protein abundance in RCC cells was detected by Western blot after treatment of cullin-based ubiquitin E3 ligase inhibitor MLN4924 (1 μM) for 48 h, or proteasome inhibitor MG132 (10 μM) for 12 h. Figure S6: Construction and verification of miR-224-5p stably expressed RCC cells and EVs. (A) Protein levels of cyclin D1 and PD-L1 in miR-224-5p stable expressed RCC cells were detected by Western blot. (B) PD-L1 protein abundance in RCC-NC/miR-224-5p cells was detected by Western blot after transfection of NC (200 nM) and miR-224-5p (100 or 200 nM) inhibitors. (C) EV uptake assays were conducted by confocal microscope. Cell membrane, EVs and nucleus were labeled with Dil (red), PKH67 (green) and DAPI (blue), respectively. Scale bar, 100 μm. Figure S7. Original Western blots of Figure 1C, Figure 5D, Figure 6A,D, Figure 7B–D, Figures S4, S5B,C, and S6A,B. Table S1: Specimen information of urinary EVs. Table S2: Specimen information of tissues of RCC patients.
