# Supplementary material for: miR-224-5p Contained in Urinary Extracellular Vesicles Regulates PD-L1 Expression by Inhibiting Cyclin D1 in Renal Cell Carcinoma Cells
Source: Cancers (Basel). 2021 Feb 4;13(4):618. doi: 10.3390/cancers13040618 (PMC7913995; doi:10.3390/cancers13040618)
Supplement: Supplementary file 1 [file cancers-13-00618-s001.pdf]

Supplementary Materials

Supplemental figures

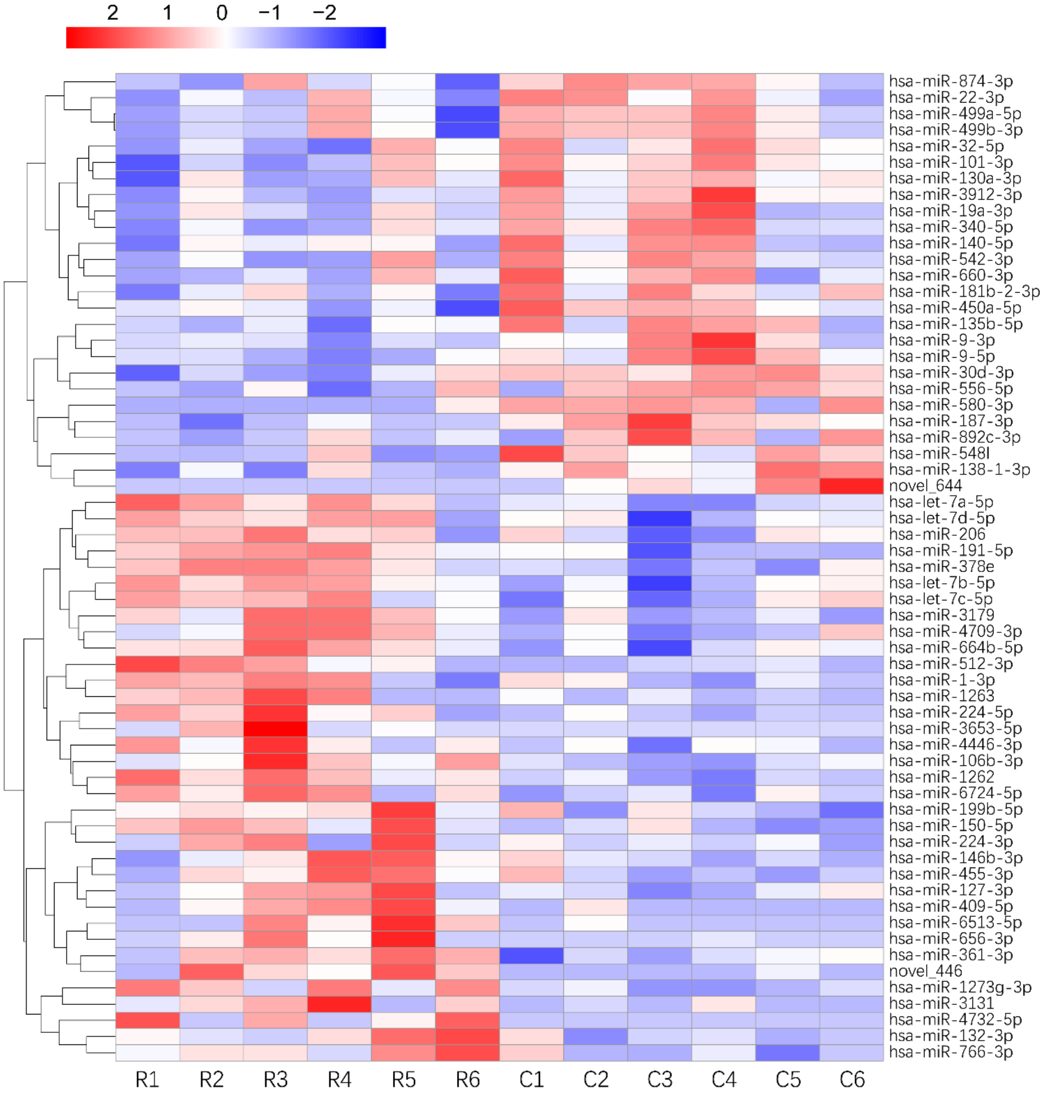

Figure S1. Heatmap of the 61 miRNAs differentially expressed in urinary EVs of RCC patients and healthy volunteers ($p < 0.05$).

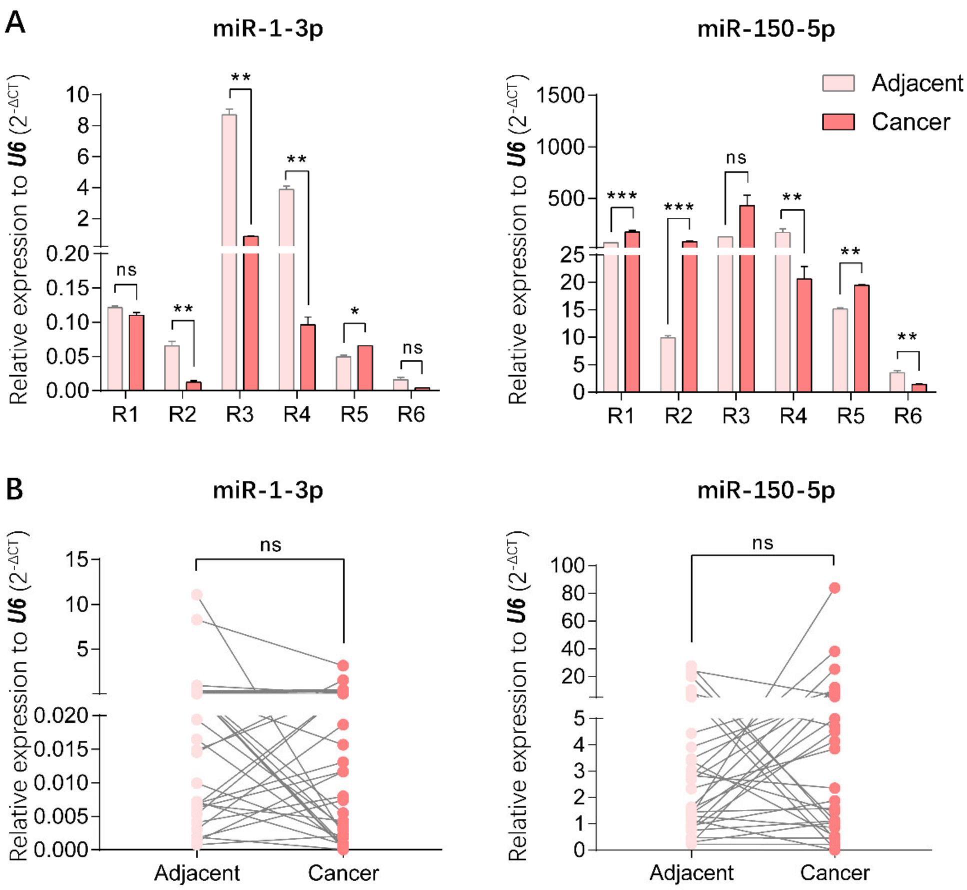

Figure S2. Expression levels of miR-1-3p and miR-150-5p in tissues of RCC patients. (A) Expression levels of miR-1-3p and miR-150-5p in cancer and adjacent tissues of RCC samples used in small RNA-sequencing were determined by RT-qPCR. (B) Expression levels of miR-1-3p and miR-150-5p in paired tissues of RCC patients were determined by RT-qPCR (n = 30). * $p < 0.05$; ** $p < 0.01$; *** $p < 0.001$; ns, no significant difference.

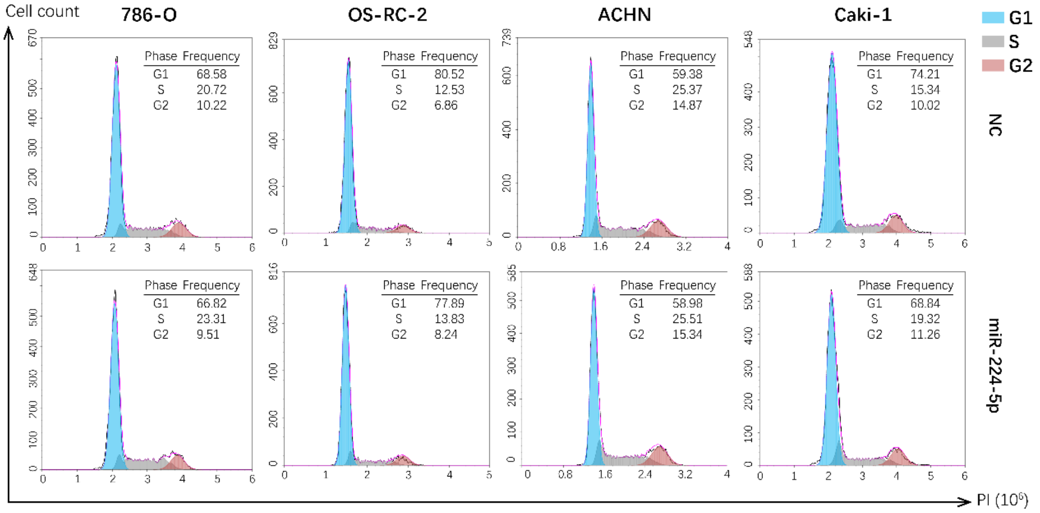

Figure S3. Cell cycle distributions after transfection of miR-224-5p inhibitors into RCC cells were determined by flow cytometry.

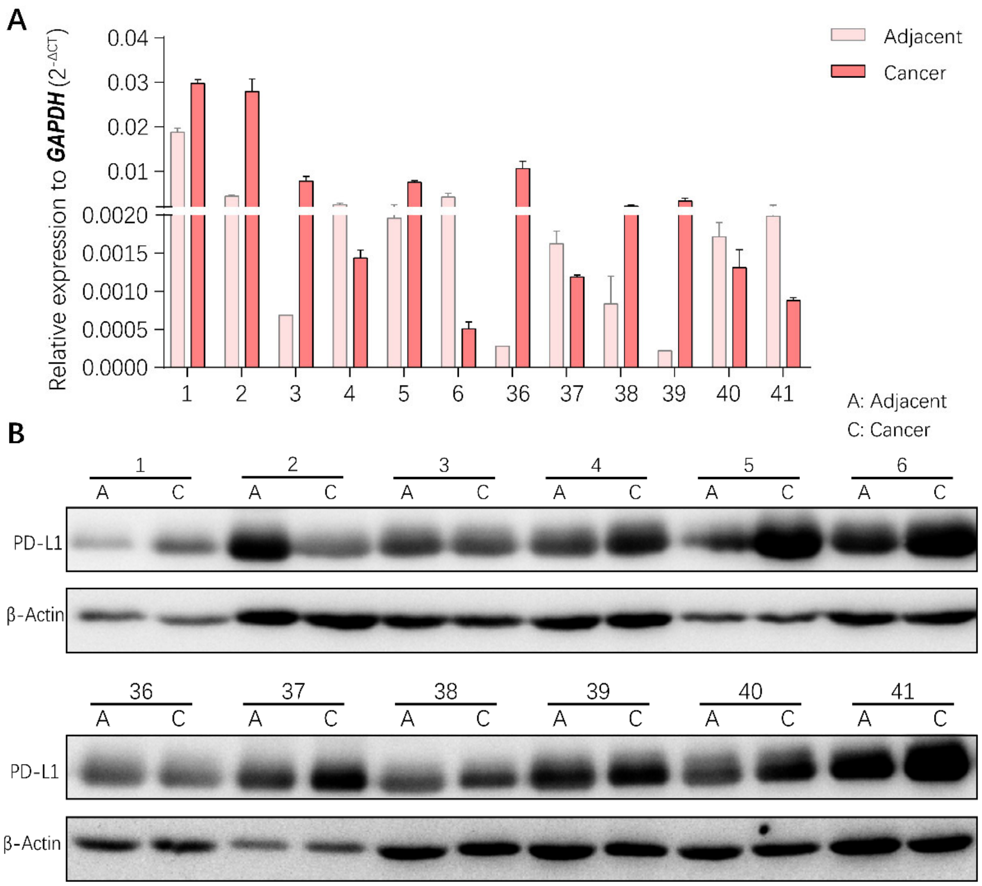

Figure S4. PD-L1 mRNA (**A**) and protein (**B**) expression levels of in paired adjacent and cancer tissues of 12 RCC patients were determined by RT-qPCR and Western blot, respectively.

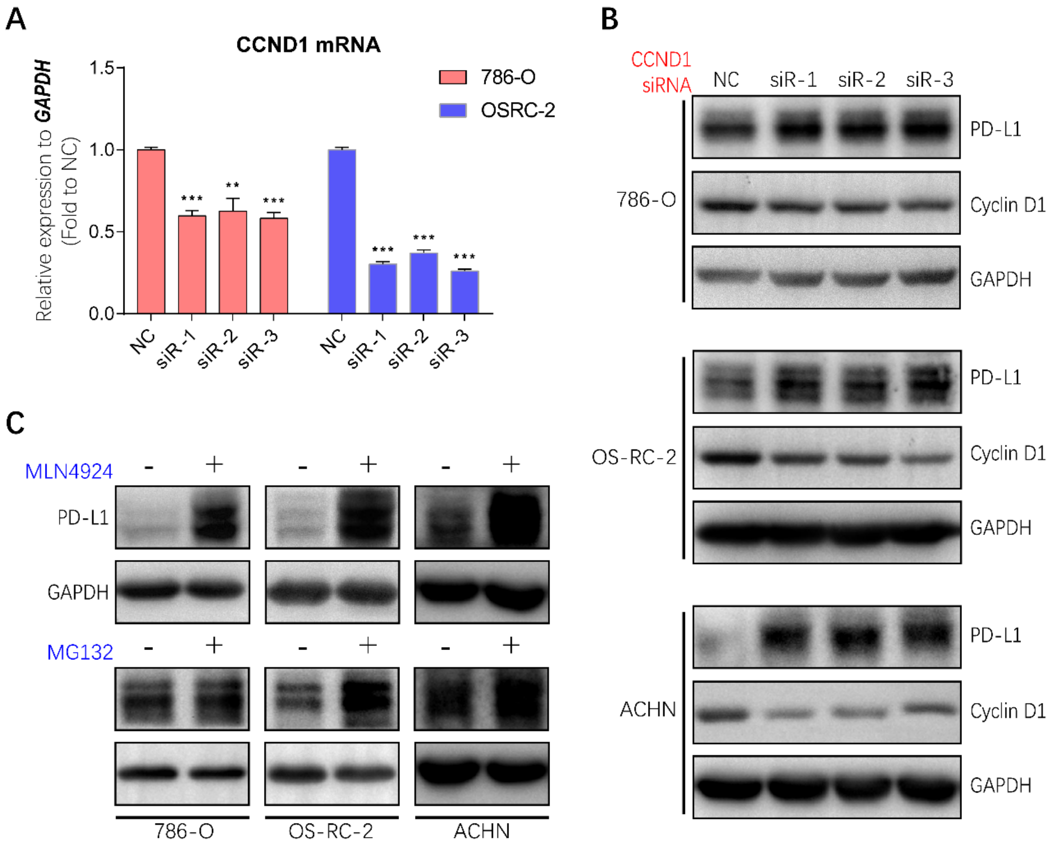

Figure S5: Regulation of PD-L1 protein expression by cyclin D-CDK4/6 and cullin 3-SPOP E3 ligase mediated proteasome degradation. (**A**) CCND1 mRNA levels in RCC cells were determined by RT-qPCR after transfection of siRNAs targeting *CCND1* (siR-1, siR-2, and siR-3). (**B**) PD-L1 protein abundance in RCC cells were detected by Western blot after transfection of siRNAs targeting *CCND1*. (**C**) PD-L1 protein abundance in RCC cells were detected by Western blot after treatment of cullin-based ubiquitin E3 ligase inhibitor MLN4924 (1 μM) for 48 h, or proteasome inhibitor MG132 (10 μM) for 12 h.

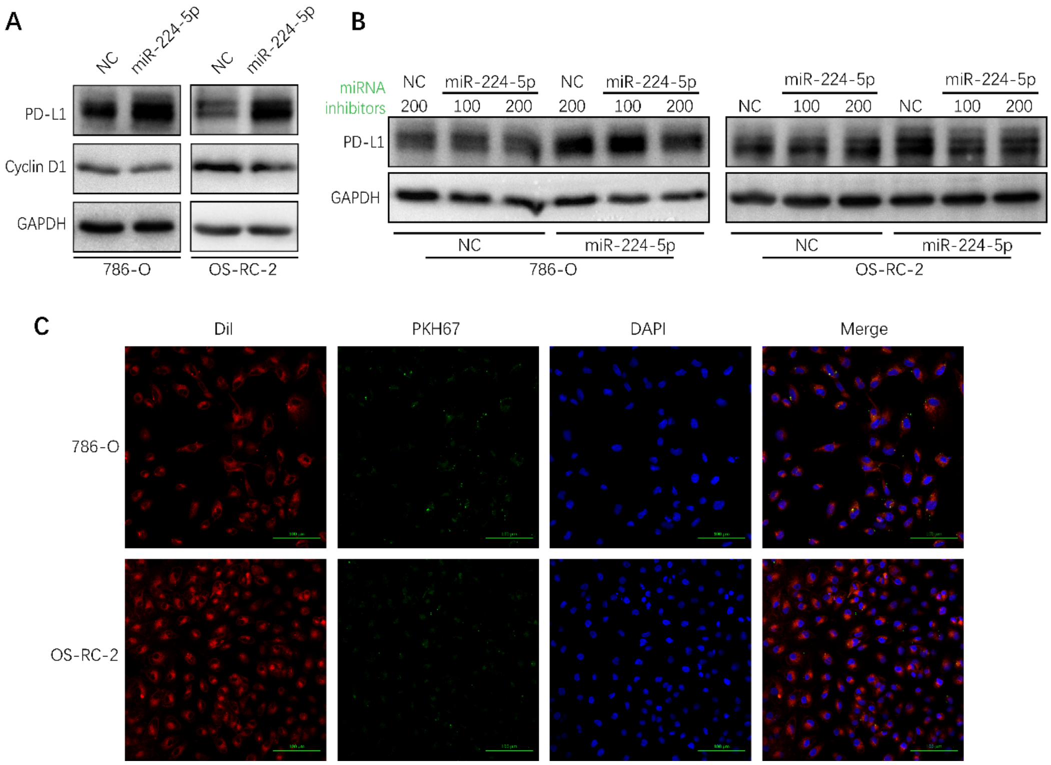

Figure S6. Construction and verification of miR-224-5p stably expressed RCC cells and EVs. (**A**) Protein levels of cyclin D1 and PD-L1 in miR-224-5p stable expressed RCC cells were detected by western blot. (**B**) PD-L1 protein abundance in RCC-NC/miR-224-5p cells were detected by Western blot after transfection of NC (200 nM) and miR-224-5p (100 or 200 nM) inhibitors. (**C**) EVs uptake assay were detected by confocal microscope. Cell membrane, EVs and nucleus were labeled with Dil (red), PKH67 (green) and DAPI (blue), respectively. Scale bar, 100 μm.

Figure 1C

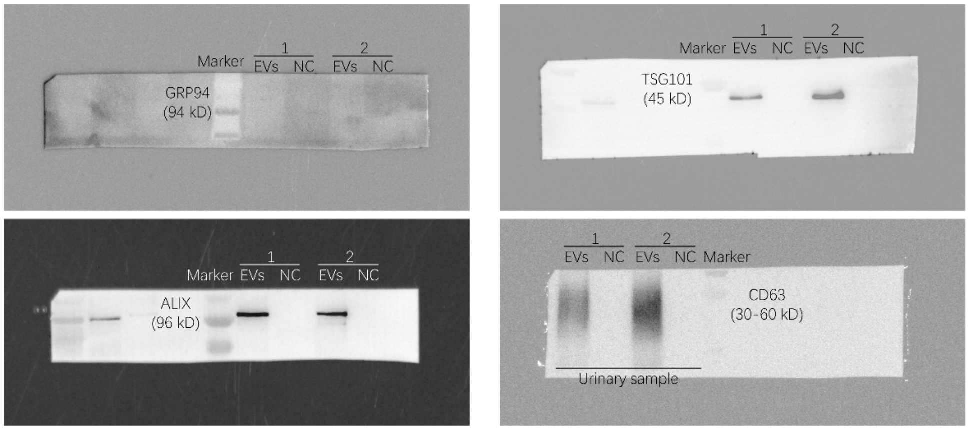

Figure 5D

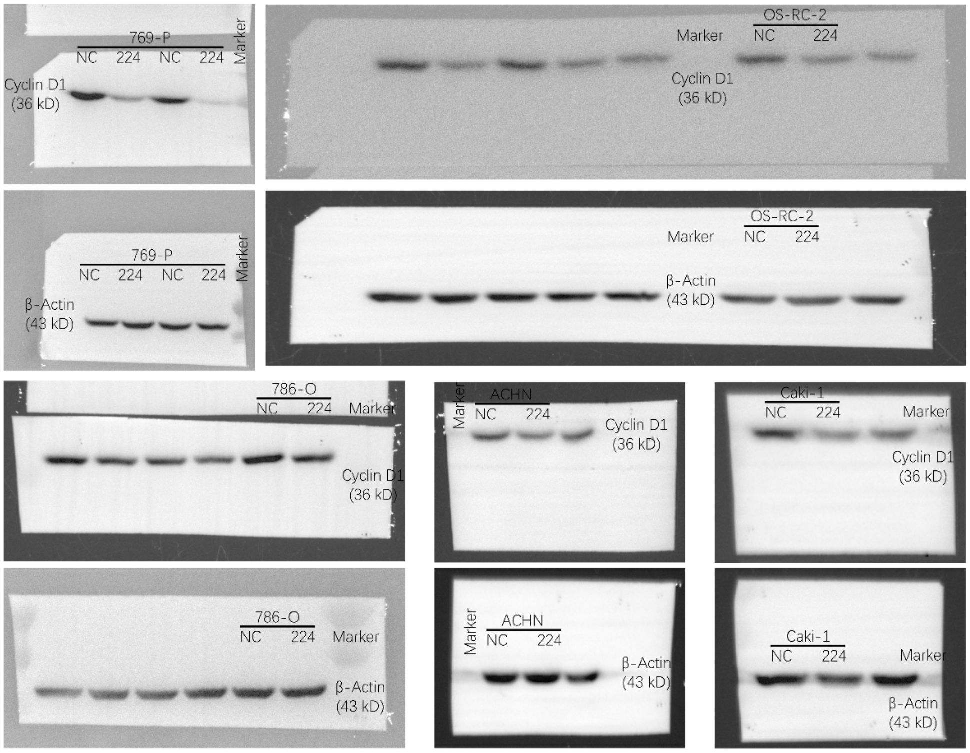

Figure 6A

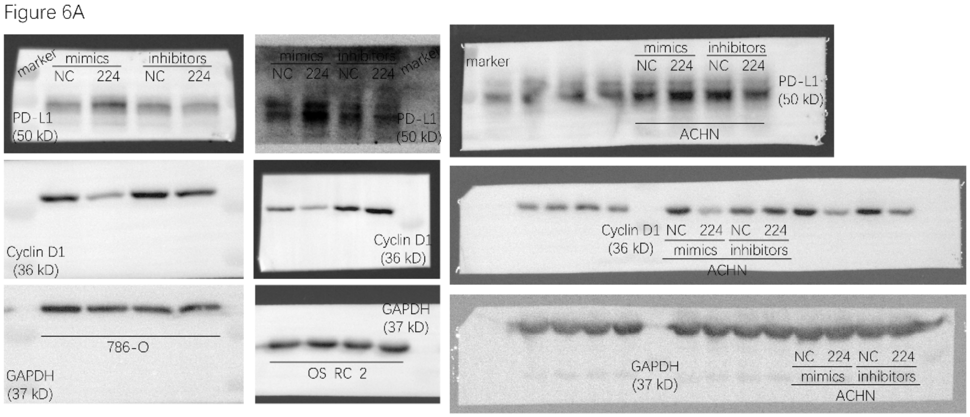

Figure 6D

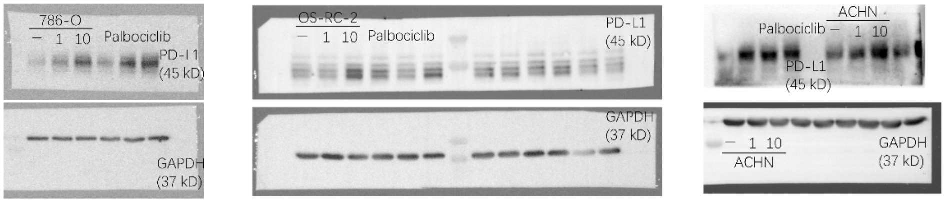

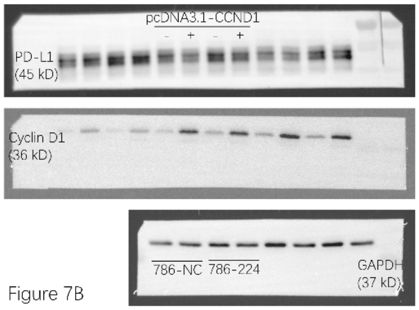

Figure 7B

Figure 7D

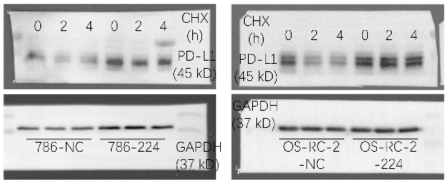

Figure 7C

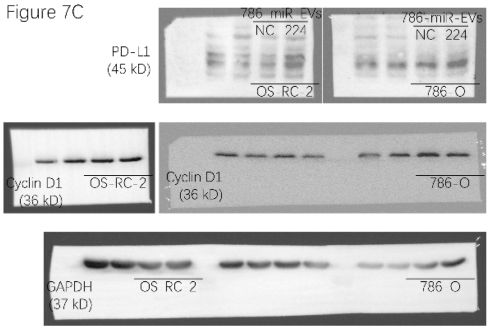

Figure S4

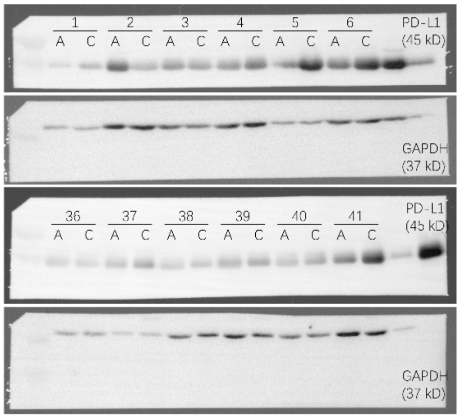

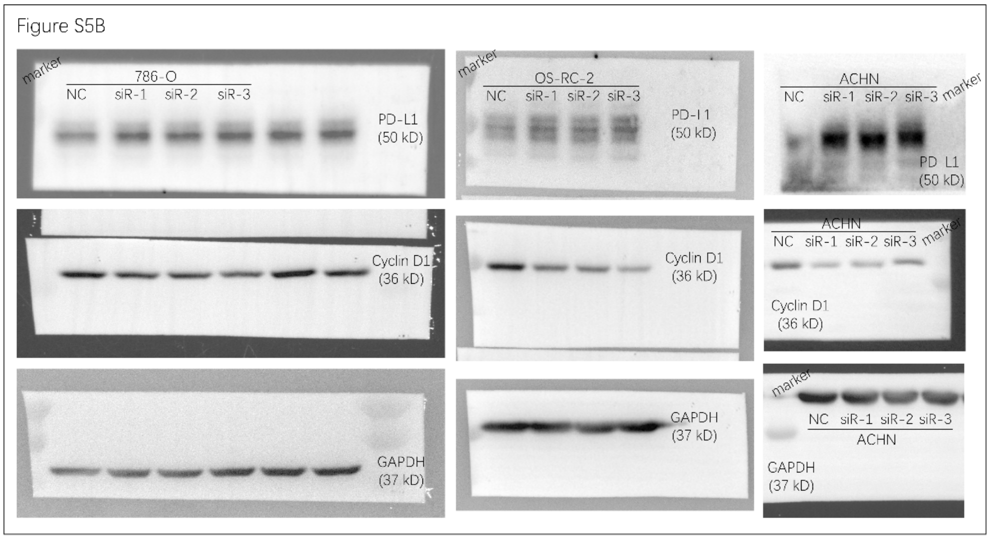

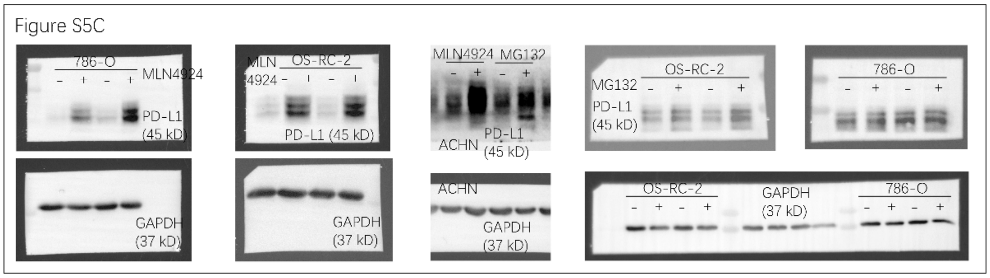

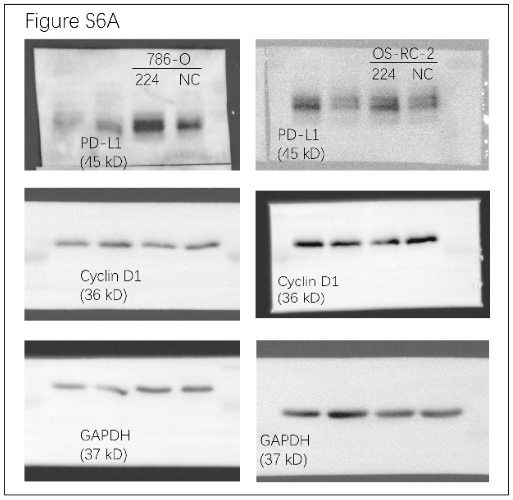

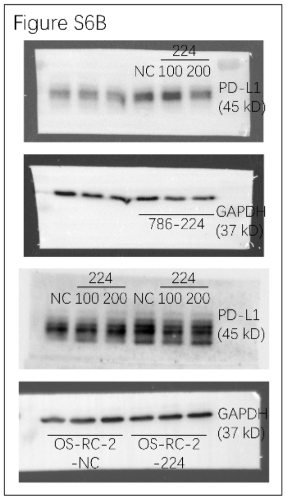

Figure S7. Original Western blots of Figure 1C, 5D, 6A, 6D, 7B, 7C, 7D, S4, S5B, S5C, S6A, and S6B.

Specimens information

Table S1. Specimens information of urinary EVs

No.	No. in figures	Gender	Age	Subtype	TNM stage
1	C1	Female	46	Healthy control subject	/
2	C2	Male	54	Healthy control subject	/
3	C3	Female	48	Healthy control subject	/
4	C4	Male	51	Healthy control subject	/
5	C5	Male	51	Healthy control subject	/
6	C6	Male	54	Healthy control subject	/
7	R1	Male	63	Clear cell renal cell carcinoma	T1bN0M0
8	R2	Male	82	Clear cell renal cell carcinoma	T1bN0M0
9	R3	Male	53	Clear cell renal cell carcinoma	T1bN0M0
10	R4	Male	51	Clear cell renal cell carcinoma	T2aN0M0
11	R5	Female	47	Clear cell renal cell carcinoma	T1bN0M0
12	R6	Male	67	Clear cell renal cell carcinoma	T1aN0M0

Table S2. Specimens information of tissues of RCC patients

No.	No. in figures	Gender	Age	Subtype	TNM stage
1	7	Female	56	Chromophobe renal cell carcinoma	T1bN0M0
2	8	Male	53	Clear cell renal cell carcinoma	T3N0M0
3	9	Male	52	Clear cell renal cell carcinoma	T1aN0M0
4	10	Male	50	Advanced renal cell carcinoma	T1bN0M0
5	11	Female	70	Chromophobe renal cell carcinoma	T1bN0M0
6	12	Female	48	Clear cell renal cell carcinoma	T1aN0M0
7	13	Male	53	Chromophobe renal cell carcinoma	T1bN0M0
8	14	Male	58	Renal oncocytoma	T1aN0M0
9	15	Male	58	Clear cell renal cell carcinoma	T1aN0M0
10	16	Male	56	Clear cell renal cell carcinoma	T2aN0M0
11	17	Male	57	Clear cell renal cell carcinoma	T1aN0M0
12	18	Male	77	Clear cell renal cell carcinoma	T1aN0M0
13	19	Male	62	Clear cell renal cell carcinoma	T1aN0M0
14	20	Male	67	Clear cell renal cell carcinoma	T1aN0M0
15	21	Male	70	Clear cell renal cell carcinoma	T3N1M0
16	22	Male	57	Clear cell renal cell carcinoma	T1aN0M0
17	23	Female	44	Clear cell renal cell carcinoma	T1aN0M0
18	24	Male	50	Clear cell renal cell carcinoma	T1bN0M0
19	25	Female	47	Clear cell renal cell carcinoma	T1aN0M0
20	26	Male	56	Clear cell renal cell carcinoma	T1bN0M0
21	27	Male	50	Clear cell renal cell carcinoma	T1aN0M0
22	28	Male	52	Clear cell renal cell carcinoma	T1aN0M0
23	29	Male	36	Clear cell renal cell carcinoma	T2bN0M0

24	30	Male	42	Clear cell renal cell carcinoma	T1bN0M0
25	31	Male	50	Clear cell renal cell carcinoma	T1aN0M0
26	32	Male	52	Clear cell renal cell carcinoma	T1aN0M0
27	33	Male	33	Clear cell renal cell carcinoma	T1aN0M0
28	34	Male	52	Clear cell renal cell carcinoma	T1aN0M0
29	35	Male	50	Clear cell renal cell carcinoma	T1bN0M0
30	55	Male	55	Clear cell renal cell carcinoma	T1bN0M0
31	36	Female	68	Clear cell renal cell carcinoma	T2aN0M0
32	37	Female	49	Clear cell renal cell carcinoma	T1bN0M0
33	38	Male	49	Clear cell renal cell carcinoma	T1bN0M0
34	39	Male	70	Clear cell renal cell carcinoma	T2aN0M0
35	40	Male	71	Clear cell renal cell carcinoma	T1bN0M0
